# Structural insights into activation mechanisms on NADase of the bacterial DSR2 anti-phage defense system

**DOI:** 10.1126/sciadv.adn5691

**Published:** 2024-07-31

**Authors:** Hong Zhang, Yu Li, Lanlan Li, Lifei Chen, Chunhua Zhu, Lifang Sun, Panpan Dong, Dingding Jing, Jinbo Yang, Lei Fu, Fangnan Xiao, Ningshao Xia, Shaowei Li, Qingbing Zheng, Yunkun Wu

**Affiliations:** ^1^ Provincial University Key Laboratory of Cellular Stress Response and Metabolic Regulation and Fujian Key Laboratory of Developmental and Neural Biology, College of Life Sciences, Fujian Normal University, Fuzhou 350117, PR China.; ^2^ State Key Laboratory of Vaccines for Infectious Diseases, Xiang An Biomedicine Laboratory, School of Life Sciences, School of Public Health, Xiamen University, Xiamen 361102, PR China.; ^3^ National Institute of Diagnostics and Vaccine Development in Infectious Diseases, Collaborative Innovation Center of Biologic Products, National Innovation Platform for Industry-Education Integration in Vaccine Research, Xiamen University, Xiamen 361102, PR China.; ^4^ Institute of Animal Husbandry and Veterinary Medicine, Fujian Academy of Agricultural Sciences, Fuzhou 350013, PR China.

## Abstract

As a sirtuin (SIR2) family protein, defense-associated sirtuin2 (DSR2) has been demonstrated to participate in bacterial anti-phage resistance via depleting nicotinamide adenine dinucleotide (NAD^+^) of infected cells, which can be activated by tail tube protein (TTP) and inhibited by DSR anti-defense 1 (DSAD1) of diverse phages. However, the regulating mechanism remains elusive. Here, we determined the cryo–electron microscopy structure of apo DSR2, as well as the respective complex structures with TTP and DSAD1. Structural analyses and biochemical studies reveal that DSR2 forms a tetramer with a SIR2 central core and two distinct conformations. Monomeric TTP preferentially binds to the closed conformation of DSR2, inducing conformational distortions on SIR2 tetramer assembly to activate its NADase activity. DSAD1 combines with the open conformation of DSR2, directly or allosterically inhibiting TTP activation on DSR2 NAD^+^ hydrolysis. Our findings decipher the detailed molecule mechanisms for DSR2 NADase activity regulation and lay a foundation for in-depth understanding of the DSR2 anti-phage defense system.

## Teaser

Unveiled bacterial DSR2 with diverse phage protein partners starts telling details of their love-hate story.

## INTRODUCTION

As the most abundant biological entities on earth, bacteriophages infect bacteria to achieve own survival and reproduction, adversely affecting bacterial growth (*1*–*3*). To resist phage infection, bacteria have evolved a series of complex defense systems, including restriction-modification systems (*4*), clustered regularly interspaced short palindromic repeats–associated systems (*5*), bacteriophage exclusion systems (*6*), abortive infection systems (*7*–*12*), and recently identified defense-associated sirtuin (DSR) defense systems (*13*, *14*).

Harboring a conservative N-terminal sirtuin (SIR2) domain, DSR proteins belong to an ancient SIR2 family (*13*). SIR2 family proteins are found in all three domains of life. In eukaryotes, SIR2 functions typically as protein deacetylases or ADP ribosyltransferases by utilizing nicotinamide adenine dinucleotide (NAD^+^) as a cofactor for the enzymatic reaction (*15*). As suggested, SIR2 proteins play important roles in various biological processes, including transcription repression, gene silencing, DNA repair, metabolic homeostasis, life-span extension, and cell cycle (*16*–*19*). In bacteria, a series of proteins containing SIR2 domains have been demonstrated to participate in anti-phage defense systems, including Thoeris (*20*–*22*), prokaryotic argonautes (pAgos) (*23*, *24*), antiviral adenosine triphosphatases (ATPases)/nucleoside triphosphatases of the signal transduction ATPases with numerous associated domains superfamily (*12*), DSR1, DSR2, and other additional systems (*13*, *25*–*28*). Short pAgos from *Geobacter sulfurreducens* with NAD^+^-bound Sir2 proteins (SIR2-APAZ) can form a stable Sir2/Ago complex and trigger endogenous NAD^+^ depletion to cause cell death upon recognizing invading plasmid or phage DNA (*24*). The Thoeris system is composed of two core proteins, ThsA and ThsB (*13*, *20*). Phage infection can trigger ThsB protein to produce an isomer of cyclic ADP-ribose, which binds to the Smf/DarA-LOG-like domain of ThsA and activates robust NADase activity of its SIR2 domain to deplete NAD^+^ of the infected cell and lastly leads to abortive infection (*20*–*22*).

DSR2 is a minimal defense system of a single protein with additional domains unidentified other than the conserved N-terminal SIR2 domain (*13*, *25*). To defend phage invasion, *Bacillus subtilis* utilizes DSR2 proteins to sense and recognize phage SPR tail tube protein (TTP), then unleashes NADase activity of its SIR2 domain to deplete the cell of NAD^+^ and abort phage propagation during infection (*25*). To counteract DSR2 abortive infection, some phages can express proteins to repress DSR2 NADase activation, such as DSR anti-defense 1 (DSAD1) from DSR2-resistant phages Phi3T and SPbeta (*25*).

The role of DSR2 in the anti-phage defense with the SIR2 domain serving as effector NADase is well established; however, the underlying molecular mechanism is too complex to be well understood. Here, using cryo–electron microscopy (cryo-EM), we determined the apo structure of DSR2 from *B. subtilis 29R* and complex structures, respectively, with TTP and DSAD1. By multiple structural comparisons combined with biochemical and mutagenesis studies, we deciphered the detailed molecular mechanisms for DSR2 activation or inhibition in response to TTP or DSAD1 from a diverse set of phages. These findings lay a solid basis for further in-depth understanding of regulation on DSR anti-phage defense systems.

## RESULTS

### DSR2 overall structure has two states of protomer in a tetrameric assembly

**Fig. 1.**
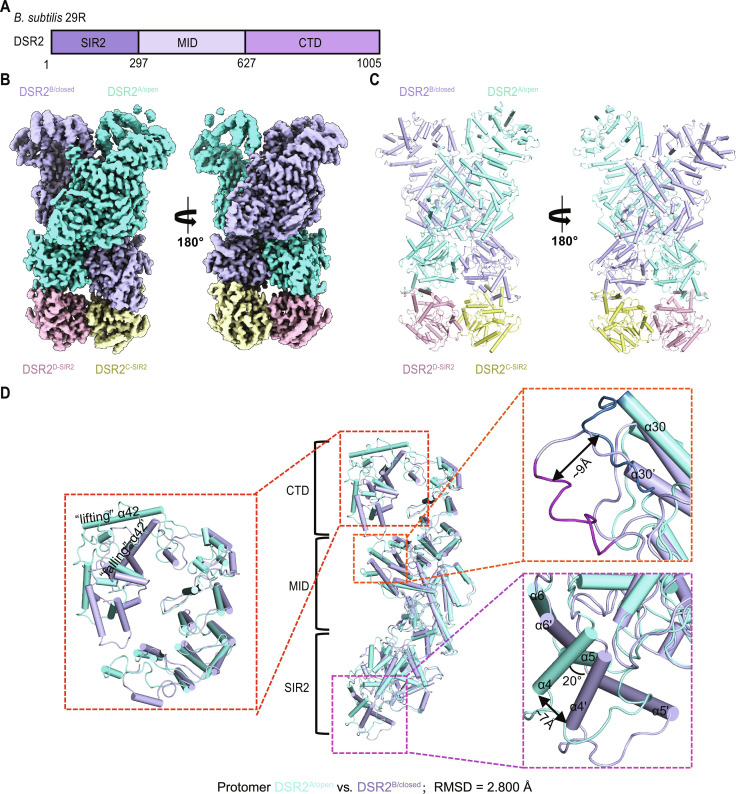
Cryo-EM analysis of apo DSR2. (**A**) Schematic domain organization of DSR2 used in this study. (**B** and **C**) Cryo-EM density map (B) and cartoon (C) representations of the 2.92-Å cryo-EM structure of the apo DSR2 tetramer containing DSR2^A^, DSR2^B^, DSR2^C-SIR2^, and DSR2^D-SIR2^ are shown. DSR2^A^, DSR2^B^, DSR2^C-SIR2^, and DSR2^D-SIR2^ are colored aquamarine, light blue, pale yellow, and light pink, respectively. (**D**) Structural comparison of protomer DSR2^A/open^ and DSR2^B/closed^.

The cryo-EM structures of DSR2 from *B. subtilis 29R* were determined in its apo form at 2.92 Å and its complexed form with TTP and DSAD1 at 3.16 Å and 2.98 Å, respectively. The final models contain only partial tetramer including two complete protomers DSR2^A^ and DSR2^B^, and two SIR2 domains of DSR2^C-SIR2^ and DSR2^D-SIR2^ due to its instability. However, DSAD1 appears to stabilize DSR2. With reference to a 6.99-Å density map of an intact DSR2-DSAD1 complex (figs. S1 to S6), it is clear that the overall structure of DSR2 forms a tetramer. The tetramer adopts a dimer-of-dimer (DSR2^AB^ and DSR2^CD^) assembly, forming an elongated dumbbell-shaped architecture with a dimension of 245 Å in length and 110 Å in width (fig. S7A). The DSR2^AB^ and DSR2^CD^ are roughly centrosymmetric. Each DSR2 protomer contains three domains, an N-terminal SIR2 domain (SIR2, residues 1 to 297), a middle connector domain (MID, residues 297 to 627), and a C-terminal sensor domain (CTD, residues 627 to 1005) (Fig. 1A).

In the structure, four N-terminal SIR2 domains form a central core consisting of two SIR2 dimers via a head-to-head arrangement with a buried interface area of ~1700 Å^2^ (Fig. 1, B and C). The complete protomer DSR2^A^ and DSR2^B^ form a scissors-like dimer with a total buried area of ~4000 Å^2^.

Akin to many well-known SIR2-containing proteins, the N-terminal SIR2 domain of DSR2 comprises a classical Rossmann-like fold and an additional triangular protrusion (α4 to α6, residues 58 to 108) (*29*). Seven parallel β strands (β3, β4, β2, β1, β5, β6, and β7) sandwiched by two helical layers form Rossmann-like fold with helices α3, α7, α8, and α14 on one side and helices α9 to α13 on the other (fig. S7B).

The MID can be divided into three parts. The first part consists of helices α15 to α19 and strands β8 and β9, forming an overall twittered ball-like shape spirally tending backward the N-terminal SIR2 domain. α15 is directly connected to α14 via a loop. Following α16 are the two anti-parallel β strands (β8 and β9). The second part contains helices α20 to α26, connected to the first part with a long loop. In the third part, helices α27 to α30 arrange almost parallel, forming a helical bundle almost perpendicular to the second part (fig. S7C).

The CTD takes a spiral arrangement that is much looser than that of the MID, implying the flexible property for sensing different phage signaling molecules. Helix α42 (residues 879 to 902) has two distinct conformations, “lifting” for DSR2^A^ and “falling” for DSR2^B^, corresponding to two states, “open” and “closed” for DSR2 protomers (fig. S7D and Fig. 1D).

Further conformational comparison indicates substantial difference between DSR2^A/open^ and DSR2^B/closed^ with a root mean square deviation (RMSD) of 2.800 Å. Besides the abovementioned identifiable α42, another obvious difference exists in the loop (α30-α31 loop, residues 633 to 643) of CTD with a ~9-Å displacement between two protomers when superimposed. In addition, the small triangular protruding module of SIR2 domain has a conformational change with a rotation of ~20° and a translation of ~7 Å (Fig. 1D).

### The phage SPR monomeric TTP activates DSR2 NADase activity

**Fig. 2.**
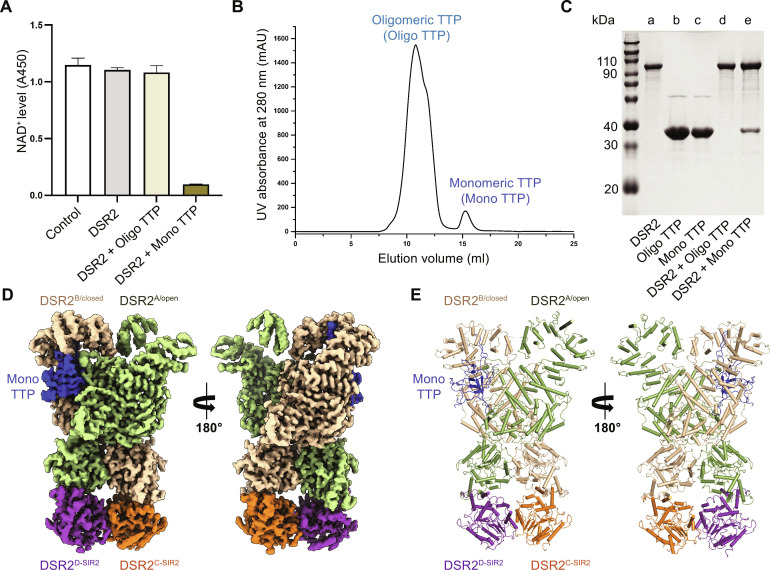
The monomeric phage SPR TTP directly binds and actives the NADase activity of DSR2. (**A**) The NADase activity of 5 μM DSR2 protein alone was compared to its activity induced by 10 μM Oligo TTP or Mono TTP. Control: The amount of NAD^+^ has not been consumed in the reaction system. (**B**) Purified TTP analyzed by size exclusion chromatography. (**C**) His-tag pulldown assays of His-tag DSR2 with oligomeric or monomeric TTP protein (Oligo TTP or Mono TTP). (**D** and **E**) Cryo-EM density map (D) and cartoon (E) representations of the 3.16-Å cryo-EM structure of the DSR2-TTP complex are shown in two different orientations. The DSR2^A/open^, DSR2^B/closed^, DSR2^C-SIR2^, and DSR2^D-SIR2^ are colored smudge, wheat, orange, and violet purple, respectively.

Studies have demonstrated that the SIR2 domain exhibits NADase activity to hydrolyze NAD^+^ and causes abortive infection in various phage resistance systems (*20*, *21*, *24*–*26*). To investigate the NADase hydrolytic activity of DSR2, DSR2 protein and substrate NAD^+^ were added with a ratio of 1:10 to the 37°C reaction system and the remaining amount of substrate was measured after 1 hour of incubation. The result indicates that DSR2 lacks the capacity to catalyze NAD^+^ hydrolysis alone, suggesting a potential self-inhibitory property of apo DSR2 (Fig. 2A).

Phage SPR TTP has been suggested to activate the *B. subtilis 29R* DSR2 anti-phage defense system (*25*). To verify this, TTP was expressed in *Escherichia coli*, and the cell lysate was purified. During SEC purification, TTP was collected in an oligomeric state (Oligo TTP) and a monomeric state (Mono TTP), respectively (Fig. 2B). His-tag pulldown experiments performed by incubating His-tag DSR2 protein with Oligo TTP and Mono TTP, respectively, on ice for 2 hours demonstrate that only Mono TTP is capable of binding with DSR2 (Fig. 2C). Furthermore, in vitro NADase activity assays obviously indicated that only the Mono TTP, not the Oligo TTP, can activate the NADase activity of DSR2 (Fig. 2A).

### DSR2-TTP complex structure indicates monomeric TTP binding preference to DSR2^closed^

**Fig. 3.**
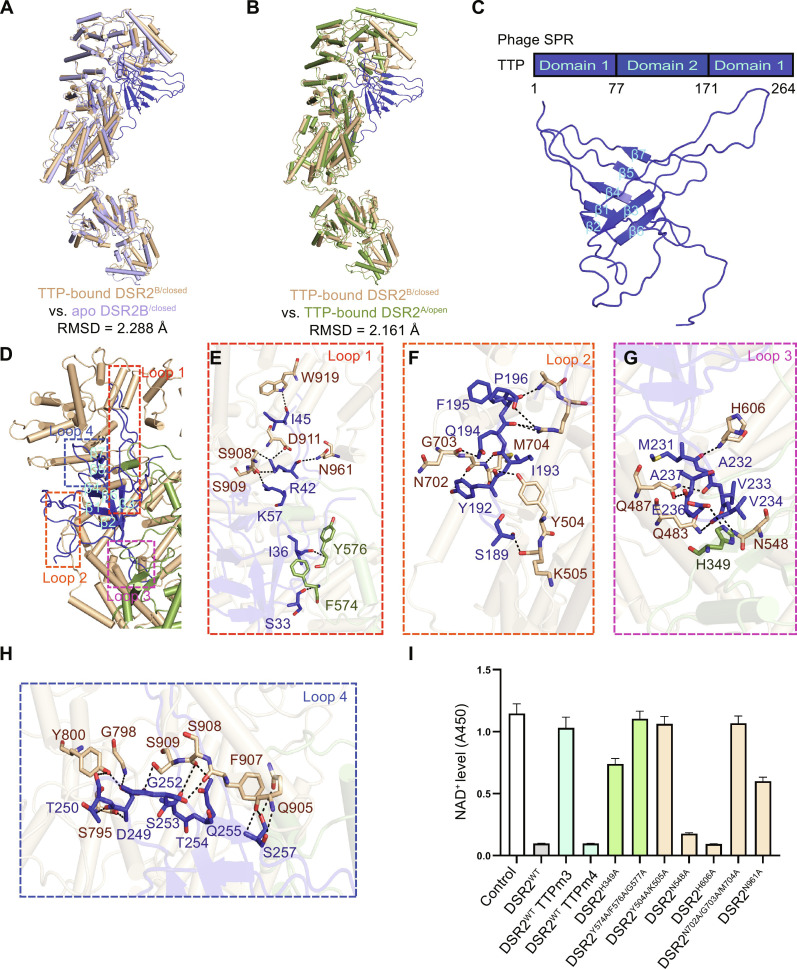
Monomeric TTP preferentially binds to the closed conformation of DSR2. (**A** and **B**) Structural comparison shows TTP-bound DSR2^B/closed^ with apo DSR2^B/closed^ (A) and TTP-bound DSR2^A/open^ (B). (**C**) Schematic domain organization of phage SPR TTP and cartoon representation of TTP (Domain 1, residues 1 to 73 and 189 to 263). (**D**) Interface analysis between DSR2 and TTP. (**E** to **H**) Detailed views of loop 1 (E), loop 2 (F), loop 3 (G), and loop 4 (H) of TTP involved in binding with DSR2. (**I**) The NADase activity assay results were obtained for wild type of DSR2 (DSR2^WT^) induced by TTP mutations (TTPm3: M231A/V233A/V234A/E236A or TTPm4: D249A/T250A/G252A/S253A/T254A/Q255A/S257A) and DSR2^WT^ or DSR2 mutations induced by wild type of TTP.

The determined cryo-EM structure of the DSR2-TTP complex contains DSR2^A/open^, DSR2^B/closed^, and SIR2 domains of DSR2^C^ (DSR2^C-SIR2^) and DSR2^D^ (DSR2^D-SIR2^), and one TTP monomer bound only to DSR2^B/closed^, instead of DSR2^A/open^ (Fig. 2, D and E). To elucidate the rationale behind TTP binding preference to the closed state DSR2 protomer, we superimposed the protomer of TTP-bound DSR2^B/closed^ onto both apo DSR2^B/closed^ and TTP-bound DSR2^A/open^ (Fig. 3, A and B). By examining the key helix α42 that distinguishes open and closed states for DSR2 protomers, clearly α42 of TTP-bound DSR2^closed^ takes the position very similar to that of apo DSR2^closed^, instead of DSR2^open^. Definitely, the closed pocket on the DSR2^closed^ should be more suitable for accommodating TTP (Fig. 3, A and B).

A top-ranked TTP model predicted through AlphaFold2 with a mean predicted local distance difference test (pLDDT) score of 85.4 suggests that TTP can be divided into two domains connected by two flexible loops (residues 78 to 85 and residues 157 to 170). Domain 1 contains residues 1 to 77 and 171 to 264, and domain 2 contains the remaining residues (*30*, *31*) (fig. S8). In the complex structure, owing to the remarkable flexibility of domain 2, only TTP domain 1 with residues 1 to 73 and 189 to 263 was determined (Fig. 3C). Domain 1 encompasses strands β1 to β7, which make extensive interactions with DSR2^closed^ with a buried area of ~2900 Å^2^. The interfaces of TTP involved in binding with DSR2 can be categorized into four loops: the β2-β3 loop (loop 1) including residues S33, I36, R42, I45, and K57; the loop preceding β4 (loop 2) including residues S189, Y192, I193, Q194, F195, and P196; the β5-β6 loop (loop 3) including residues M231, A232, V233, V234, E236, and A237; and the loop following β7 (loop 4) including residues D249, T250, G252, S253, T254, Q255 and S257. Notably, loop 1 inserts into the CTD and loop 3 extends across the CTD to penetrate even to the MID, indicating that these two loops may play key roles in TTP binding and activation on DSR2 NADase activity (Fig. 3, D to H).

To determine their key roles in activating DSR2, mutagenesis studies on TTP have been designed with the abovementioned key residues of the four TTP loops mutated to alanine, respectively. Failing to obtain the monomeric proteins of mutated loop 1 and loop 2, only monomeric loop 3 and loop 4 mutants (TTPm3 and TTPm4) were used to conduct DSR2 NADase hydrolysis experiments in vitro. Coincident with the above-described structural observation, TTPm3 substantially reduces the NADase activity, whereas TTPm4 mutant basically has no effect (Fig. 3I). In addition, alanine mutations of the key interface residues in DSR2, including H349, Y504/K505, Y574/F576/G577, N702/G703/M704, and N961, impaired the activation of NADase activity, also suggesting the notable importance of TTP binding for the activation of DSR2 NADase activity (Fig. 3I).

### Conformational distortion on SIR2 tetramer assembly is crucial for NADase activity

**Fig. 4.**
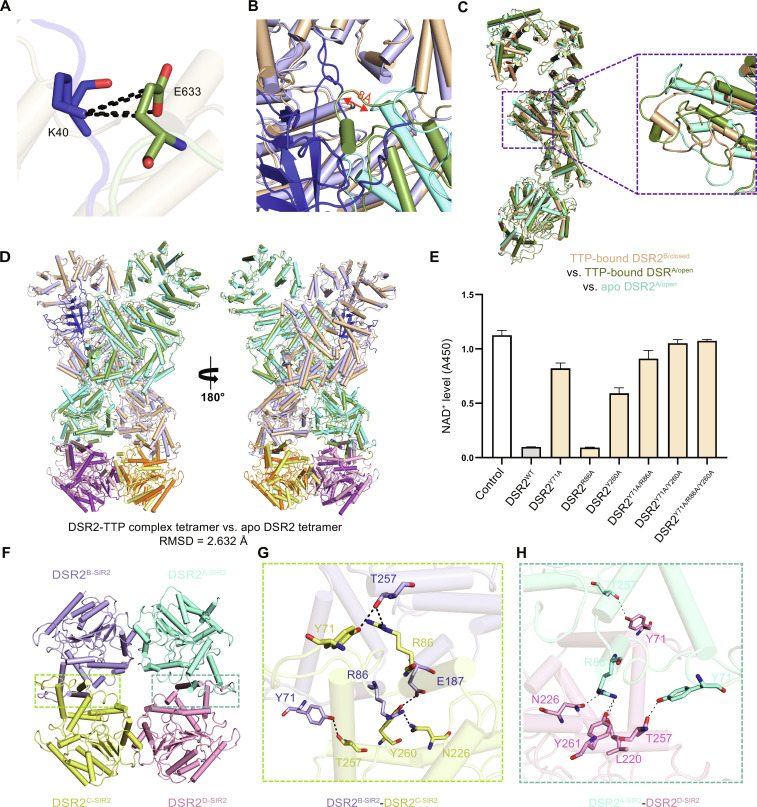
The crucial role of SIR2 domains’ conformational distortions in the DSR2 tetramer assembly for NADase activity. (**A**) Representation shows the hydrogen bond and salt bridge between residues K40 of TTP and residues E633 of TTP-bound DSR2^A/open^. (**B**) A detailed view of the loop (residues 633 to 643) in TTP-bound DSR2^A/open^ displacing toward TTP is provided. (**C**) Structural comparison of TTP-bound DSR2^B/closed^ with TTP-bound DSR2^A/open^ and apo DSR2^A/open^. (**D**) Structural comparison between the DSR2-TTP complex tetramer and the apo DSR2 tetramer is conducted. (**E**) The NADase activity assay results of DSR2^WT^ and DSR2 mutants induced by TTP are shown. (**F** to **H**) The tetramerization of SIR2 domains is illustrated (F), including the interaction displays between DSR2^B-SIR2^ and DSR2^C-SIR2^ (G), as well as between DSR2^A-SIR2^ and DSR2^D-SIR2^ (H).

As described above, the binding pocket on CTD of DSR2^B/closed^ widens to accommodate TTP with only a small switch of its helix α42. However, the bound TTP makes contact with the adjacent DSR2^A/open^, forming a hydrogen bond and a salt bridge with the α30-α31 loop of DSR2^A^ CTD (Fig. 4A). Notably, this interaction drags the loop of DSR2^A/open^ toward TTP with a distance of 8 Å (Fig. 4B). This dragged position is almost the one of DSR2^B/closed^, implying a transition state of DSR2^A^ conformational change triggered by TTP from an open state to a closed state (Fig. 4C).

The combination of TTP acts like a key, allosterically causing the MID and SIR2 domain of both protomers to undergo profound conformational changes with complicated translocation of its secondary structure elements, though the conformational changes that happened on DSR2^B^ and DSR2^A^ are quite different (Fig. 4D). As the core of the DSR2 tetramer, the SIR2 tetramer takes a dimer-of-dimer form with a head-to-head arrangement. Through superimposition comparisons between protomers with or without TTP, the overall assembly of the SIR2 tetramer shows that distinct rotations also happened on the SIR2 domains of DSR2^C^ and DSR2^D^ (Fig. 4D). Thus, combining with in vitro NADase hydrolysis analyses, this conformational change with a large distortion of the SIR2 domain tetramer induced by TTP is suggested to play an important role on triggering the NADase activity of DSR2 (Fig. 4E).

Furthermore, at the interfaces of the SIR2 tetramer, residues Y71 from each SIR2 domain form hydrogen bonds with residues T257 from the adjacent SIR2. Residue R86 of DSR2^A-SIR2^ interacts with residues L220, N226, and Y261 of DSR2^D-SIR2^, respectively. Residues R86, E187, and T257 of DSR2^B-SIR2^ form hydrogen bonds with residues N226, Y260, and R86 of DSR2^C-SIR2^, respectively (Fig. 4, F to H). On the basis of the interactions, mutations including Y71A, R86A, Y260A, Y71A/R86A, Y71A/Y260A, and Y71A/R86A/Y260A have been introduced for in vitro NADase assays. The results reveal that R86A has a negligible impact on the DSR2 activation induced by TTP; however, the mutants Y71A, Y260A, and Y71A/R86A exhibit varying degrees of abortion for the DSR2 NADase activation, and mutants Y71A/Y260A and Y71A/R86A/Y260A substantially impair DSR2 activation (Fig. 4E). Because of the key roles of these interface residues in maintaining tetramer conformation for SIR2, these mutagenesis analyses further support that appropriate conformation for SIR2 tetramerization should be essential for its NADase hydrolytic function.

### DSR2-DSAD1 complex structure shows DSAD1 binding preference to DSR2^open^

**Fig. 5.**
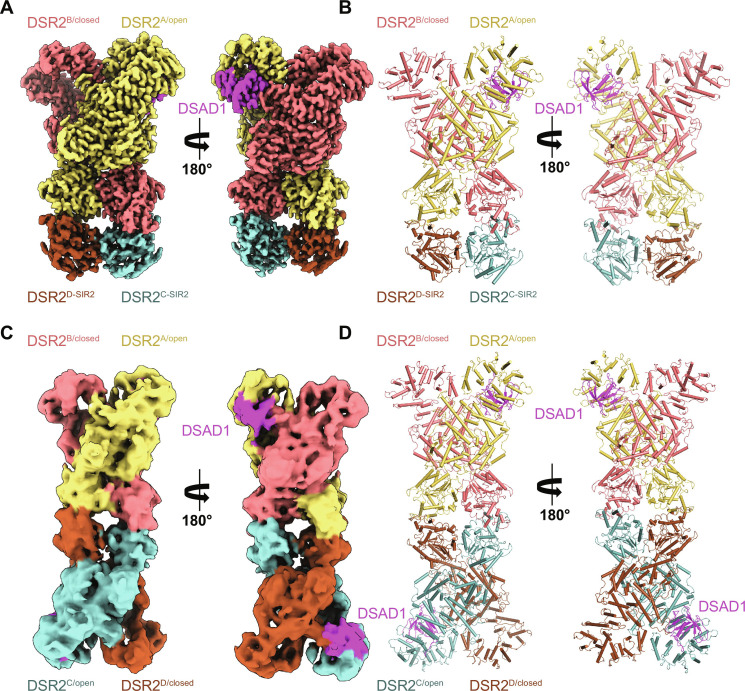
Cryo-EM structure of the DSR2-DSAD1 complex. (**A** and **B**) Cryo-EM density map (A) and cartoon (B) representations show the 2.98-Å cryo-EM structure of the DSR2-DSAD1 complex in two different orientations. DSR2^A/open^, DSR2^B/closed^, DSR2^C-SIR2^, and DSR2^D-SIR2^ are colored yellow orange, deep salmon, light teal, and brown, respectively. (**C** and **D**) Cryo-EM density map (C) and cartoon model (D) depict the intact DSR2-DSAD1 complex tetramer in two different orientations, colored in the same way as (A) and (B).

**Fig. 6.**
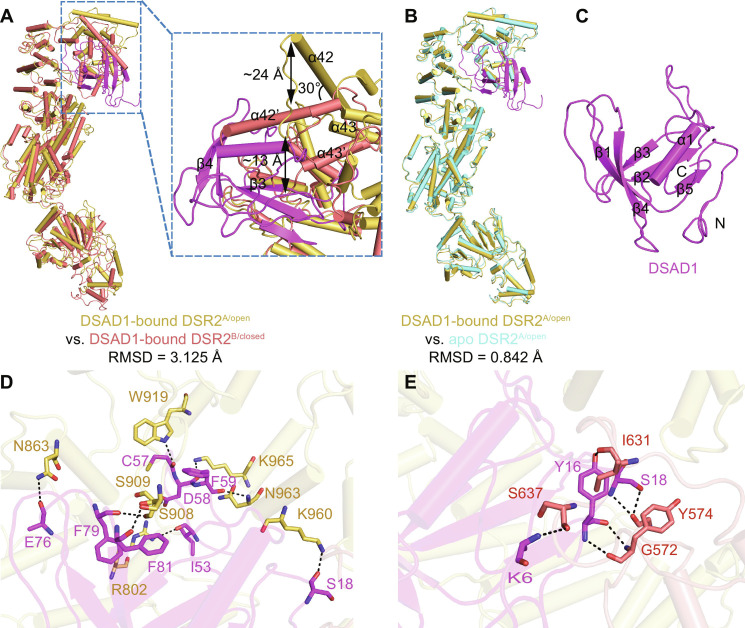
The binding preference of DSAD1 to open conformation of DSR2. (**A**) Comparison between the binding pockets of DSR2^A/open^ and DSR2^B/closed^ in the DSR2-DSAD1 complex. (**B**) Structural comparison between DSAD1-bound DSR2^A/open^ and apo DSR2^A/open^. (**C**) Structural representation of DSAD1. (**D** and **E**) Interactions of DSAD1 with DSR2^A/open^ (D) and DSR2^B/closed^ (E) in the DSR2-DSAD1 complex.

As studied, phage SPbeta DSAD1 can directly bind DSR2 and inhibit its NADase activity (*25*). To elucidate underlying mechanisms, the cryo-EM structure of the DSR2-DSAD1 complex has been determined at 2.98 Å, with DSAD1 bound to DSR2^A^ in the open state (Fig. 5, A and B). Another intact tetramer density map of the DSR2-DSAD1 complex at a resolution of 6.99 Å enables us to unequivocally establish that DSAD1 exclusively combines with the open conformation protomers of DSR2, with a binding ratio of 2:4 (Fig. 5, C and D). To decipher the binding preference of DSAD1, the comparison of the binding pocket on DSR2^A/open^ by superimposing to DSR2^B/closed^ indicates that α42 of DSR2^B/closed^ has a ~30° rotation at the pocket opening, which causes ~24 and ~13 Å falling for α42 and the α42-α43 loop, respectively, blocking the entry of DSAD1 with severe clashes to DSAD1 β4 and β3 (Fig. 6A), while the binding pocket on DSR2^A/open^ is well accommodable for DSAD1, with small conformational change upon binding when compared to apo DSR2^A/open^ (Fig. 6B).

In the structure, DSAD1 consists of strands β1 to β5 and one α helix between β4 and β5 (Fig. 6C). Extensive interactions between DSAD1 and DSR2^open^ consist of residues S18, I53, C57, D58, F59, E76, F79, and F81 of DSAD1 and residues K960, R802, W919, K965, N963, N863, S909, and S908 of DSR2^open^ (Fig. 6D). In addition, the binding of DSAD1 introduces new contact with DSR2^closed^ by DSAD1 residues K6, Y16, and S18 forming hydrogen bonds with DSR2^closed^ residues S637 and I631 of CTD and residues G572 and Y574 of the MID (Fig. 6E).

**Fig. 7.**
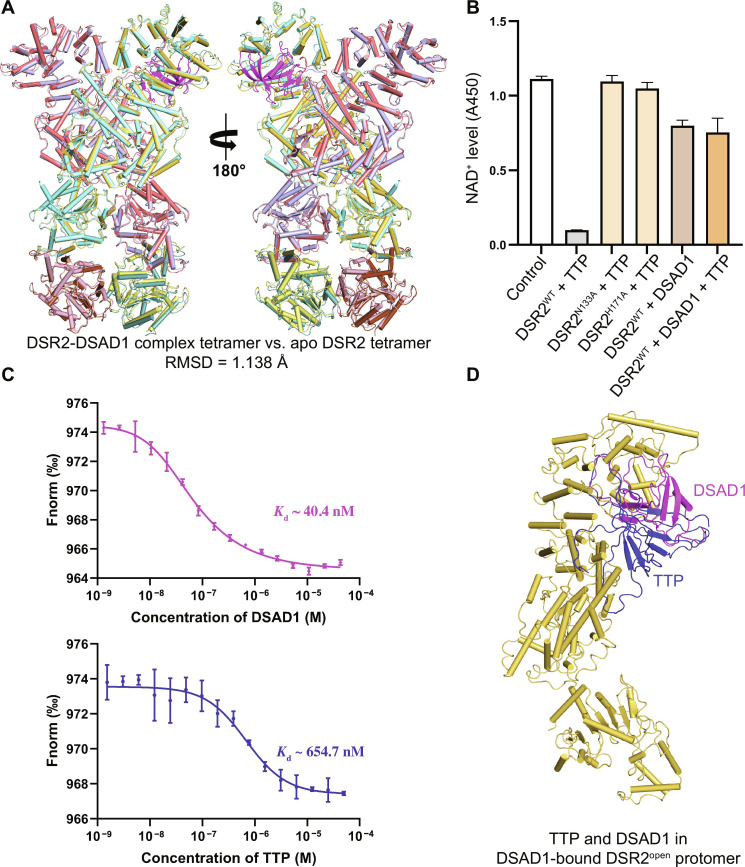
The analyses of DSAD1 binding and inhibiting DSR2 activation. (**A**) The overall structural superimposition of the DSR2-DSAD1 complex with apo DSR2. (**B**) The results of DSAD1-induced DSR2^WT^ NADase activity and TTP-induced DSR2^WT^, and DSR2 mutants, and DSAD1-bound DSR2^WT^ NADase activity. (**C**) Quantification of binding affinities between fluorescently labeled DSR2 and DSAD1/TTP by microscale thermophoresis. The experiment was performed three times. (**D**) Simultaneous presentation of superimposed TTP and DSAD1 in the DSAD1-bound DSR2^open^ protomer.

Compared to TTP binding of DSR2, the DSAD1 binding induces much slighter conformational change with an RMSD of 1.138 Å between the overall structures of the DSR2-DSAD1 complex and apo DSR2 (Fig. 7A). Although the SIR2 domains within the DSR2-DSAD1 complex exhibit conformational twisting to a certain extent, the magnitude of twist is much smaller compared to the changes induced by TTP binding. This conformational change on the SIR2 domain tetramer is not harsh enough to hurt proper tetramerization conformation critical for its NADase activity. This structural observation is also consistent with our in vitro DSR2 NADase analysis for DSAD1 regulation, which indicates that DSAD1 can induce DSR2 degrading NAD^+^ slightly (Fig. 7B).

The respective binding affinities of DSAD1 and TTP to DSR2 measured by microscale thermophoresis (MST) assays demonstrate that DSAD1 and TTP directly bind to DSR2 with dissociation constant (*K*_d_) values of 40.4 and 654.7 nM, respectively. Notably, DSAD1 exhibits a higher affinity for DSR2 compared to TTP (Fig. 7C).

## DISCUSSION

### Phage DSAD1 directly or allosterically inhibits TTP activation on DSR2 NADase activity

In many bacteria, the rapid hydrolysis of central metabolite molecules NAD^+^ has been deployed to cause cell death, thereby preventing propagation of invaders (*22*, *32*). DSR2 defense system was found to contain a conserved SIR2 domain associated with NADase activity (*13*, *25*), which achieve abortive infection through eliciting NAD^+^. Studies have shown that DSR2 can be activated by newly translated phage SPR TTP to become an active NADase, causing NAD^+^ depletion and self-killing of the infected cell to protect the bacterial population. Meanwhile, DSAD1 from phage Phi3T/SPbeta was demonstrated to bind and inactivate DSR2 (*25*).

As mentioned, except for a 6.99-Å density map of the intact DSR2-DSAD1 complex, the structures of apo DSR2 and DSR2-DSAD1 and DSR2-TTP complexes have been determined with a partial tetramer. In the negative stain images and 2D classification averages in a 680-pixel extraction box size, the complete tetrameric form for apo DSR2 and the DSR2-TTP complex is present but limited, while the particles partially appear to exhibit instances of absence or blurriness at certain terminals (figs. S1 and S2), implicating intrinsic instability or susceptibility to degradation of these regions. The specific underlying mechanisms possibly due to the sample preparation process or unidentified intrinsic factors await further investigations.

With our determined cryo-EM structures of DSR2, multiple conformational comparisons have been carefully examined by superimpositions among these DSR2 protomers from apo DSR2 and DSR2-TTP and DSR2-DSAD complexes. Two distinct states of DSR2 are revealed, with the open state preferentially recognizing DSAD1 and the closed state preferring TTP. When superimposing TTP and DSAD1 simultaneously in one protomer (Fig. 7D), DSAD1 binds to DSR2^open^ with the lifting α42 generating a wider binding pocket; the falling of α42 in the closed state clearly blocks the entry of DSAD1 like a door bolt (Fig. 6A). However, the narrowed binding pocket on DSR2^closed^ is quite suitable for TTP, which sits at a lower part of CTD and closer to the MID and forms extensive interactions with these two domains (Figs. 3, D to H, and 7D). With the direct steric hindrance in the binding site occupied by DSAD1, it is impossible for TTP to bind to the same DSR2 protomer.

In addition to binding with DSR2^open^ at the main binding pocket on CTD, DSAD1 forms hydrogen bonds with the MID of adjacent protomer in the closed state. Similarly, TTP not only interacts with the CTD binding pocket of DSR2^closed^ but also drags a loop with an ~8-Å movement from its open state position to the closed state position, implying a transition state induced by TTP binding. Thus, it is hypothesized that TTP binding might induce conformational changes of the adjacent DSR2 protomer from an open state to a closed state for TTP binding and lastly unleashes drastic NADase activity for abortive infection.

These perceptions on the CTD and MID obviously induce and transmit conformational changes to the SIR2 domain of the DSR2 tetramer, which is the active site for NADase hydrolysis. With an intact SIR2 tetramer in our three DSR2 structures, conformational changes for SIR2 tetramerization can be better compared. For the SIR2 domain tetramer, TTP binding induced larger conformational distortion than DSAD1 binding. With a role of transmitting the conformational changes from domain CTD to SIR2, the MID is crucial for TTP activation of DSR2^closed^ NADase activity. Notably, DSAD1 binding also forms strong interactions with the MID of DSR2^closed^ and therefore locking the conformation and preventing the conformational change passed on to the SIR2 tetramer upon TTP binding. It seems that TTP and DSAD1 compete with each other on regulating DSR2 NADase activity allosterically.

However, the exact activating mechanisms for degrading NAD^+^ substrate is difficult to elucidate without a determined DSR2-NAD^+^ complex structure. A modeled structure, with an estimated binding free energy of −4.85 kcal/mol, suggests that the NAD^+^ binding site is located between the Rossmann-like fold and the small triangular protrusion of the SIR2 domain, which is on the surface of the DSR2 tetramer and not adjacent to the SIR2 tetramerization interfaces (fig. S9). In addition, the active site suggests that residues N133 and H171 are crucial for DSR2 NADase activity. In vitro NADase assays further confirm both mutants of N133A and H171A aborting the NADase activity (Fig. 7B), which is consistent with the result of previously reported phage infection analyses (*25*).

**Fig. 8.**
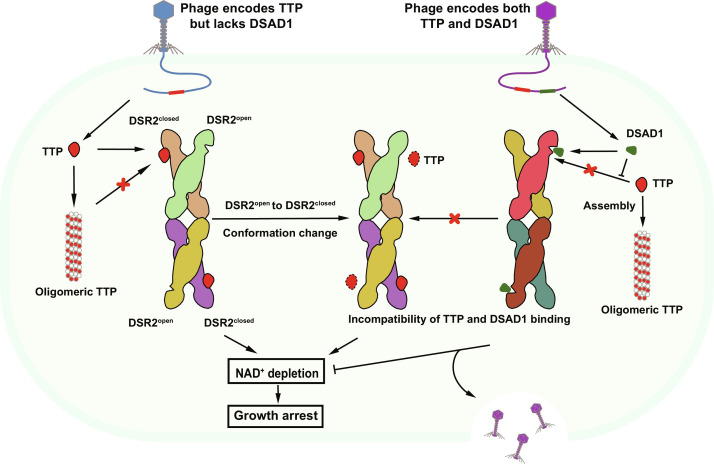
Proposed mechanistic model of DSR2 activity regulation by TTP and DSAD1.

On the basis of these structural analyses, a mechanistic model of DSR2 regulation on DSR2 NADase activity is proposed here (Fig. 8). Phage monomeric TTP is capable of binding to DSR2^closed^. The binding of TTP not only induces SIR2 domain tetramerization conformational changes of DSR2 to activate its NADase activity but also potentially triggers DSR2 open state transition to a closed state. To counteract this activation, some phages evolve to express proteins like DSAD1 to prevent this self-killing of infected bacterial cells. Structurally, DSAD1 specifically binds to DSR2^open^. The binding of DSAD1 locks DSR2^open^ in the resting state, by not only competitively occupying the binding pocket but also stabilizing the MID of the adjacent protomer to stop the transduction of TTP-induced conformational changes in DSR2^closed^, allosterically inhibiting the activation on DSR2 NADase activity triggered by TTP binding. Together, DSAD1 could directly or allosterically inhibit TTP binding and activation on DSR2 NADase activity.

Meanwhile, in vitro NADase activity assays indicate that apo DSR2 exhibits negligible NADase activity (Fig. 2A), DSAD1 itself has a weak effect on DSR2 activation, and monomeric TTP has a strong activation on DSR2 NADase activity (Fig. 7B). However, with the presence of DSAD1, TTP loses its activation property for DSR2 NADase, further confirming that DSAD1 can inhibit the activation of DSR2 NADase activity induced by TTP (Fig. 7B).

## MATERIALS AND METHODS

### Protein expression and purification

To express and purify DSR2 from *B. subtilis* (WP_029317421), DSAD1 from phage phi3T/SPbeta (WP_004399562), and TTP from phage SPR (WP_010328117), the genes encoding DSR2, DSAD1, and TTP were synthesized by Sangon Biotech. The *DSR2* gene was cloned into the pET28a plasmid, while the *DSAD1* and *TTP* genes were inserted into a modified pET32a vector. The pET32a vector can encode an N-terminal His-tag and a fused thioredoxin tag (Trx-tag), followed by a cleavage site for the tobacco etch virus (TEV) protease. The recombinant proteins were expressed in competent cells of *E. coli* BL21(DE3). Bacterial cells were cultured in LB medium with kanamycin (50 μg/ml) or ampicillin (100 μg/ml) at 37°C, and protein expression was induced by adding 0.3 mM isopropyl-d-1-thiogalactopyranoside for 16 hours when the OD_600_ (optical density at 600 nm) reached 0.6 to 0.8. Next, cells were harvested and lysed by ultrasonication on ice in a lysis buffer containing 25 mM tris-HCl, pH 8.0, 500 mM NaCl, 5% glycerol, 2 mM β-mercaptoethanol, and 0.1% Tween 20. Cell debris was separated by centrifugation at 15,000*g* for 30 min at 4°C.

The overexpressed target protein was purified from the supernatant using a Ni^2+^-NTA affinity resin column (GE Healthcare). After being washed with a washing buffer containing 25 mM tris-HCl, pH 8.0, 500 mM NaCl, and 5% glycerol supplemented with 30 mM imidazole, the target proteins were eluted by a wash buffer supplemented with 300 mM imidazole. The DSR2 protein was concentrated in the centrifugal filter (Amicon Ultra) and then purified directly using size-exclusion gel filtration with a Superdex^200^ Increase 10/300 GL column (GE Healthcare). TEV protease was used to cleave the N-terminal His-tag and Trx-tag of DSAD1 and TTP proteins, which were subsequently removed by being loaded onto a Ni^2+^-NTA column again. Afterward, the eluted protein was pooled, concentrated, and further purified using the same size-exclusion gel filtration. Last, the proteins were collected in a gel-filtration buffer containing 50 mM tris-HCl, pH 8.0, 150 mM NaCl, and 2 mM dithiothreitol.

To obtain the DSR2-DSAD1 or DSR2-TTP complex, the purified DSR2 protein was incubated with DSAD1 or TTP protein at a molar ratio of 1:3 for at least 2 hours on ice. The complex protein was then purified again using size-exclusion gel filtration.

### Site-directed mutagenesis

Plasmid pET28a-DSR2 and plasmid pET32a-TTP were used as templates. Mutations of DSR2 and TTP were generated using a Fast Mutagenesis Kit (Vazyme, C214, and C215) and confirmed by DNA sequencing. Then, all mutated plasmids were transformed into *E. coli* BL21 (DE3) competent cells for protein expression, following the same procedure as mentioned above. The primers used in this study are summarized in table S1.

### NADase activity assays in vitro

The NAD^+^ degradation assays were performed in a reaction buffer containing 50 mM tris-HCl, pH 8.0, and 150 mM NaCl. β-Nicotinamide adenine dinucleotide trihydrate (β-NAD^+^; Sangon Biotech, A600641) was used as the substrate. A reaction mixture of 100 μl was prepared, which contained a final concentration of 5 μM DSR2 (add 10 μM TTP and/or 10 μM DSAD1) and 50 μM β-NAD^+^. The reaction system was incubated at 37°C for 1 hour. Then, the reaction system was diluted by a 10-fold factor, and a volume of 20 μl from the diluted sample was immediately used to measure the remaining amount of β-NAD^+^ using the NAD^+^/NADH quantitation kit (Beyotime, S0175). All NADase activity assays in vitro were carried out at least three times. Data analysis was performed using GraphPad Prism 9.

### Cryo-EM sample preparation and data collection

Aliquots (3 μl) of mixtures (1.0 mg/ml) of either purified DSR2 protein or its complex with DSAD1 or TTP were loaded onto glow-discharged (80 s at 20 mA) holey carbon Quantifoil grids (AU 1.2/1.3, 300 mesh, Quantifoil Micro Tools) using a Vitrobot Mark IV (Thermo Fisher Scientific) at 100% humidity and 4°C. As for the samples, DSR2 complexed with DSAD1 was acquired using the EPU software on a Titan Krios G4 (Thermo Fisher Scientific) operated at 300 kV. Images were recorded using a Gatan K3 detector in a 32-frame movie mode at a nominal 130,000× magnification with a pixel size of 0.325 Å, and the total electron dose was set to 60 e^−^ Å^−2^, with an exposure time of 4.5 s.

### Image processing and 3D reconstruction

Drift and beam-induced motion correction were performed with MotionCor2 (*33*) to produce a micrograph from each movie. Contrast transfer function (CTF) fitting and phase-shift estimation were conducted with Gctf (*34*). Micrographs with astigmatism, obvious drift, or contamination were discarded before reconstruction. The following reconstruction procedures were performed using Cryosparc V3 (*35*). In brief, particles were automatically picked using the “Blob picker” or “Template picker.” Several rounds of reference-free 2D classifications were performed, and the selected “good” particles were then subjected to ab initio reconstruction, heterogeneous refinement, and final nonuniform refinement. Localized refinement focusing of the local areas was also performed if necessary. The resolutions of all density maps were determined by the gold standard Fourier shell correlation curve, with a cutoff of 0.143 (*36*). Local resolutions of maps were estimated with ResMap (*37*).

### Atomic model building, refinement, and 3D visualization

The initial models of the DSR2, DSAD1, or TTP were acquired from alphafold2 (*30*). We initially fitted the models into the corresponding final cryo-EM maps using Chimera (*38*), and further corrected and adjusted them manually by real-space refinement in Coot (*39*). The resulting models were then refined with phenix.real_space_refine in PHENIX (*40*). These operations were executed iteratively until the problematic regions, Ramachandran outliers, and poor rotamers were either eliminated or moved to favored regions. The final atomic models were validated with Molprobity (*41*, *42*). All figures were generated with PyMOL (The PyMOL Molecular Graphics System, Schrödinger), Chimera (*38*), or ChimeraX (*43*, *44*). Table S2 summarizes the model statistics.

### MST assays

The purified DSR2 proteins were fluorescently labeled using MO-L011 Monolith Protein Labeling Kit RED-NHS second generation according to the manufacturer’s instructions. A serial twofold dilution method was used to prepare TTP or DSAD1 protein with varying concentrations. Labeled DSR2 (100 nM) was mixed in equal volumes with different concentration gradients of TTP or DSAD1 protein. The mixtures were incubated for 20 min in running buffer (10 mM phosphate-buffered saline and 0.05% Tween 20) at room temperature to enable binding. The samples were loaded into capillaries (Monolith NT.115 Standard Treated Capillaries, MO-K022) and measured with a Monolith NT.115 instrument (NanoTemper Technologies). Triplicate measurements were performed, and the data were analyzed using MO.Affinity Analysis software.
