# Supplementary material for: Structural insights into activation mechanisms on NADase of the bacterial DSR2 anti-phage defense system
Source: Sci Adv. 2024 Jul 31;10(31):eadn5691. doi: 10.1126/sciadv.adn5691 (PMC11290490; doi:10.1126/sciadv.adn5691)
Supplement: Supplementary file 1 — Figs. S1 to S9 Tables S1 and S2 [file sciadv.adn5691_sm.pdf]

Science Advances
AAAS

Supplementary Materials for

Structural insights into activation mechanisms on NADase of the bacterial DSR2 anti-phage defense system

Hong Zhang *et al.*

Corresponding author: Yunkun Wu, wuyk@fjnu.edu.cn; Qingbing Zheng, qbzheng@xmu.edu.cn; Shaowei Li, shaowei@xmu.edu.cn

Sci. Adv. **10**, eadn5691 (2024)
DOI: 10.1126/sciadv.adn5691

This PDF file includes:

Figs. S1 to S9
Tables S1 and S2

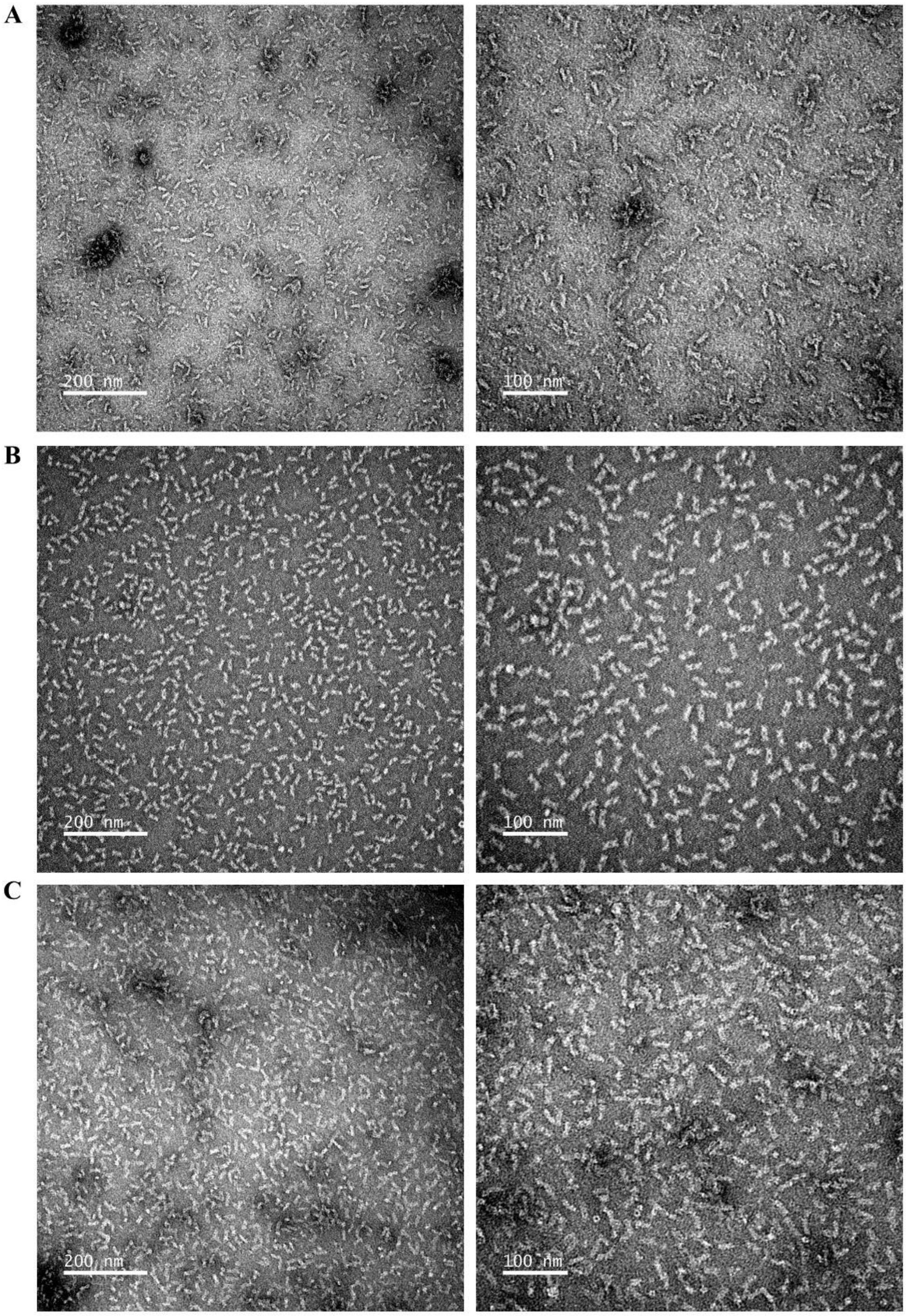

Fig. S1. Representative negative stain EM micrograph images. Representative negative stain EM micrograph images of apo DSR2 **(A)**, DSR2-DSAD1 complex **(B)**, DSR2-TTP complex **(C)**.

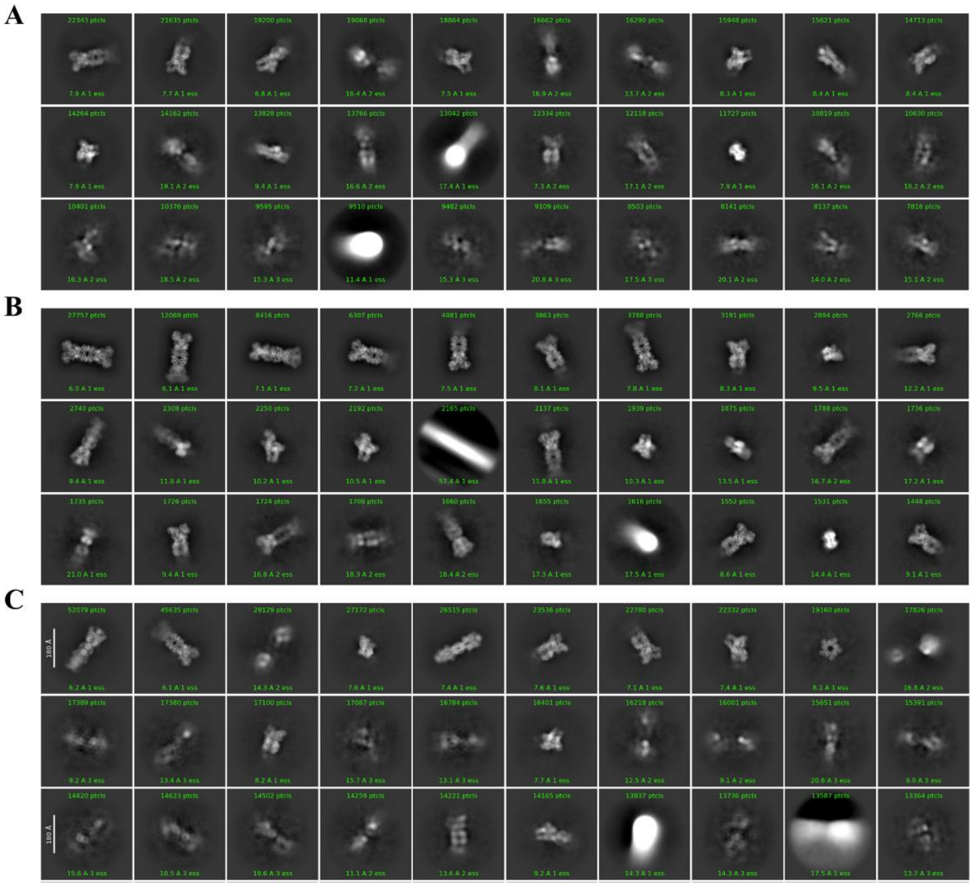

Fig. S2. The presentations of 2D classification of samples. 2D classification of apo DSR2 (**A**), DSR2-DSAD1 complex (**B**), and DSR2-TTP complex (**C**) in 680 pix extraction box size.

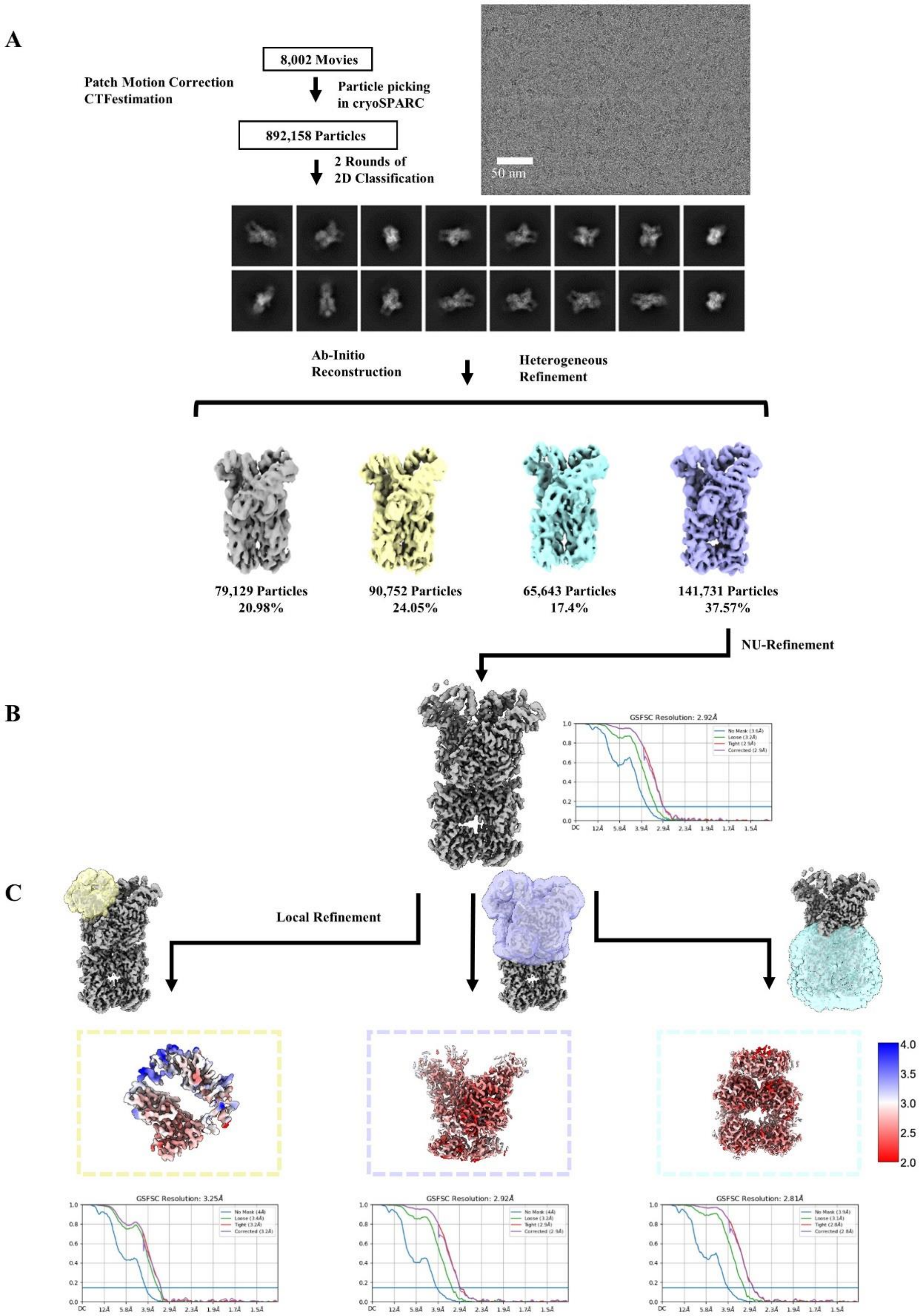

Fig. S3. Flowcharts for apo DSR2 cryo-EM data processing. (A) Representative electron micrographs, 2D classifications, initial models and final maps are shown. Scale bar: 50 nm. **(B-C)** The gold-standard FSC curves of overall maps of apo DSR2 and local refinement maps.

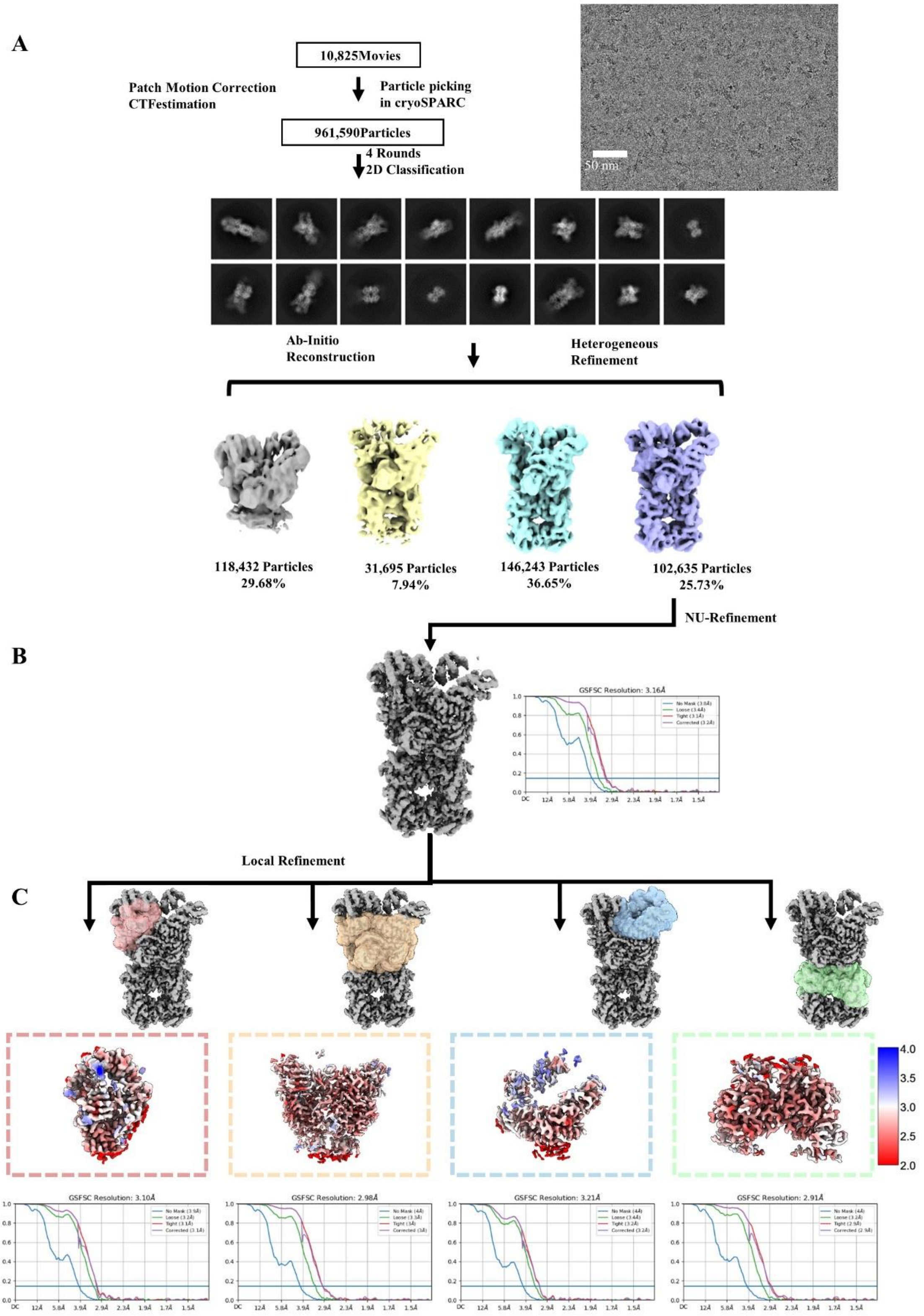

Fig. S4. Flowcharts for DSR2-TTP complex cryo-EM data processing. **(A)** Representative electron micrographs, 2D classifications, initial models and final maps are shown. Scale bar: 50 nm. **(B-C)** The gold-standard FSC curves of overall maps of DSR2-TTP complex and local refinement maps.

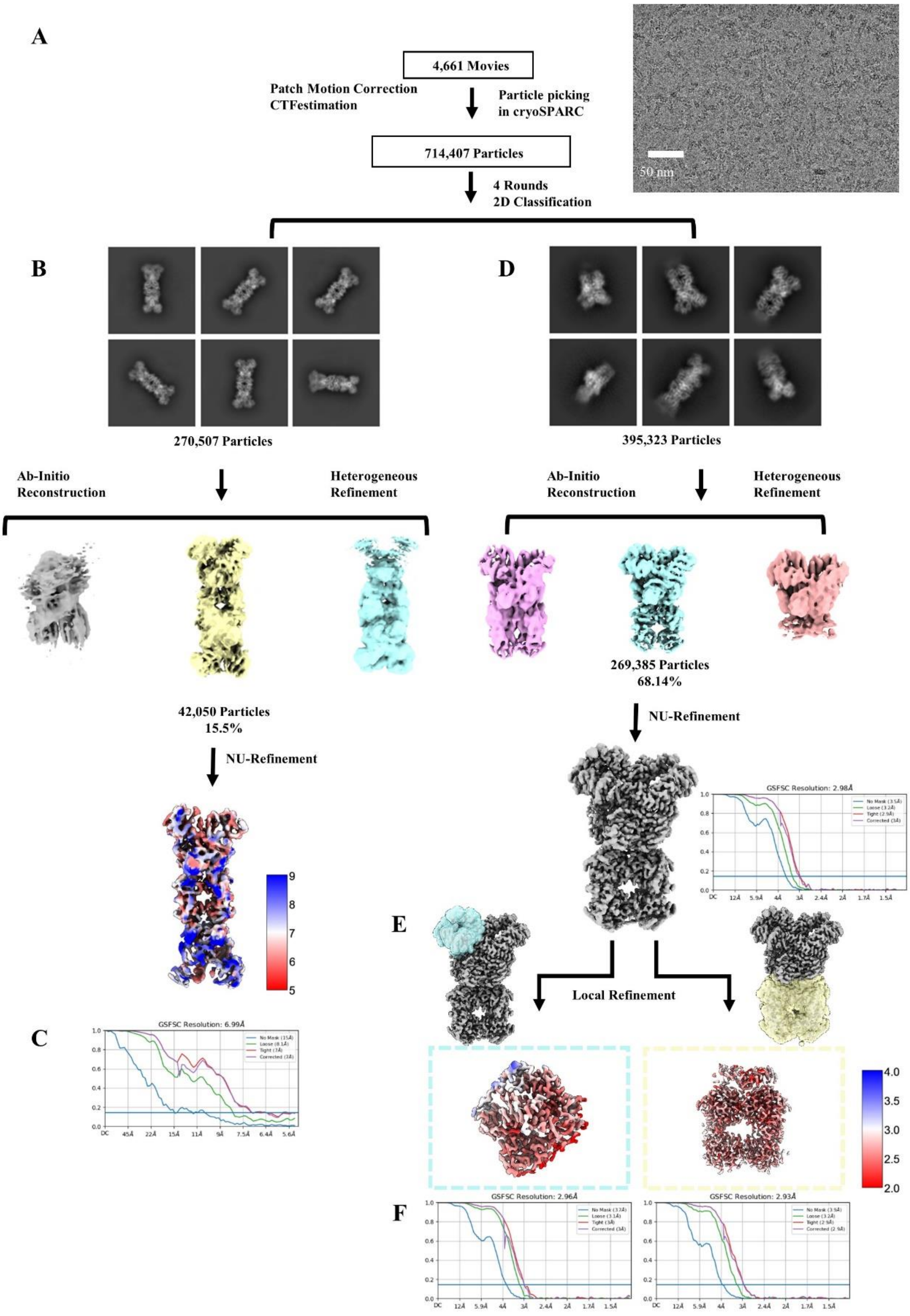

Fig. S5. Flowcharts for DSR2-DSAD1 complex cryo-EM data processing. **(A)** Representative electron micrographs are shown. Scale bar: 50 nm. **(B-C)** Intact DSR2-DSAD1 complex 2D classifications, initial models, final maps and the gold-standard FSC curves are shown. **(D-F)** Partial DSR2-DSAD1 complex 2D

classifications, initial models and final maps. The gold-standard FSC curves of overall maps of DSR2-DSAD1 complex and local refinement maps are shown.

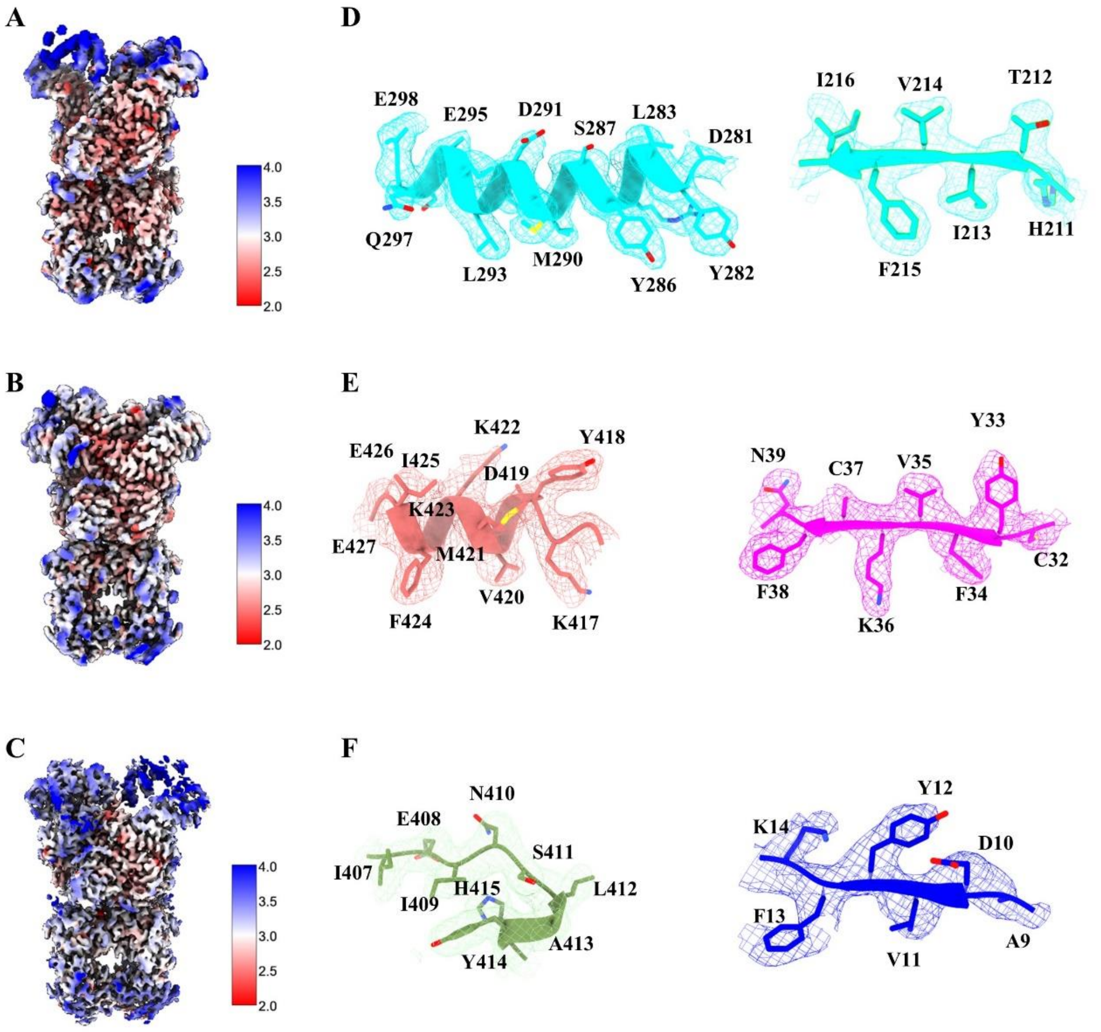

Fig. S6. Resolution estimation of the EM maps. Local resolution analysis of apo DSR2 **(A)**, DSR2-DSAD1 complex **(B)** and DSR2-TTP complex **(C)**. Representative density maps with corresponding models between apo DSR2 **(D)**, DSR2-DSAD1 complex **(E)** and DSR2-TTP complex **(F)**.

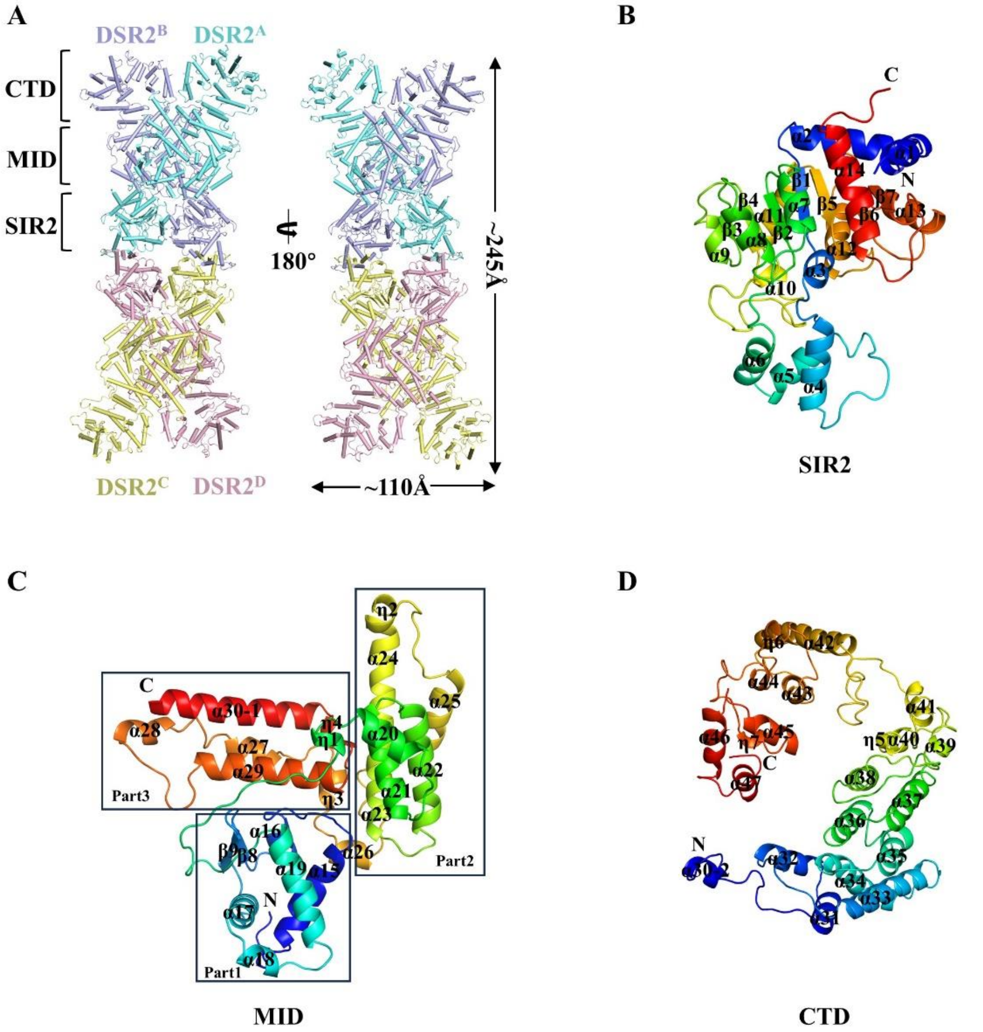

Fig. S7. Structural representations of tetramer and different domains for apo DSR2. (**A**) The overall structure of apo DSR2 tetramer (DSR2^{ABCD}) is shown in two different orientations, indicating its dimensions and height. DSR2^{A}, DSR2^{B}, DSR2^{C} and DSR2^{D} are colored in aquamarine, lightblue, paleyellow, and lightpink, respectively. Cartoon representations of SIR2 domain **(B)**, MID domain **(C)** and CTD domain **(D)**. Each domain is colored in rainbow, blue is in the N-terminal and red is in the C-terminal.

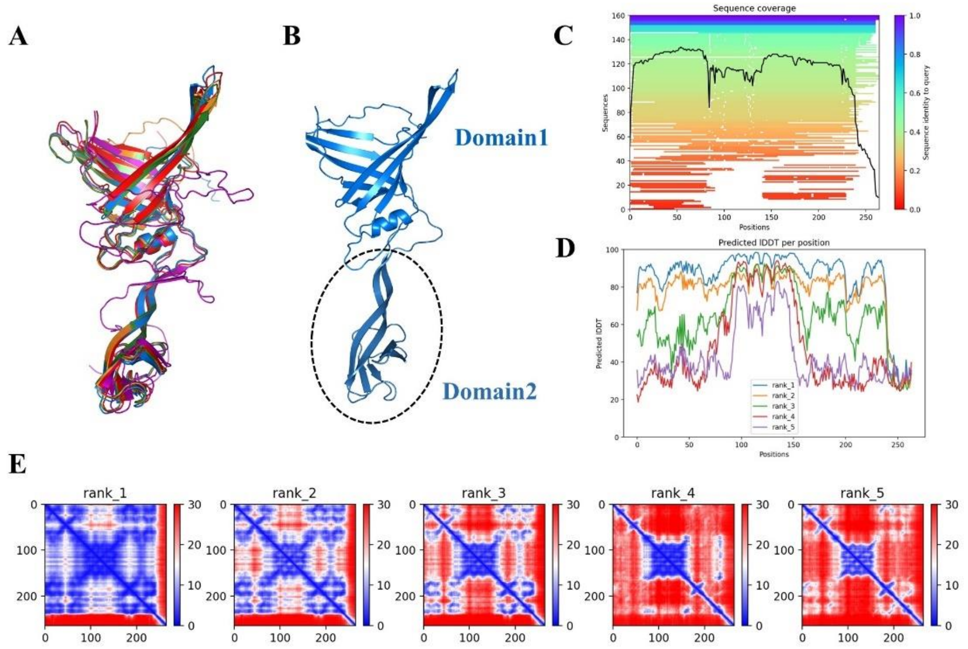

Fig. S8. The structures of phage SPR TTP predicted by Alphafold2. (A) Superimposition of the five AlphaFold predictions. **(B)** The structure of top-ranked model (rank1) contains two domains, domain1 and domain2. **(C)** Sequence coverage plot showing the number of homologues identified across the TTP sequence. A plot of the pLDDT score per position **(D)** and PAE plot **(E)** for each of the five AlphaFold predicted models.

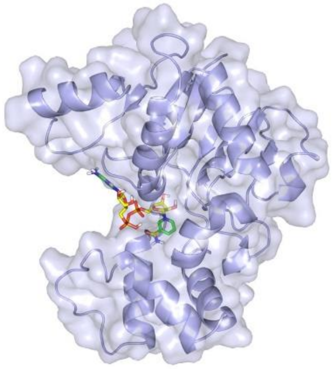

Fig. S9. The molecular docking result of the SIR2 domain with NAD^+.

Table S1. Primers used in this study.

Primer name	Sequence
**DSR2 mutagenesis**	
DSR2-Y71A-F	AGAACTGGCTGGTTCACCTAAAAAAGGTAATTATAGCT
DSR2-Y71A-R	GTGAACCAGCCAGTTCTTCATGGTATTTATCCACTAAA
DSR2-R86A-F	TCTGATGAGTATCTGGCTATCCCGCAGATTTTC
DSR2-R86A-R	GAAAATCTGCGGGATAGCCAGATACTCATCAGA
DSR2-Y260A-F	ACGAAACTCTTATAGCTTATGAAAATAAAGGTCTCCG
DSR2-Y260A-R	CGGAGACCTTTATTTTCATAAGCTATAAGAGTTTCGT
DSR2-H349A-F	GTACTGTTGTTCGTGCTAAAAATAAAGGTTTTGGTTATATGGAAC
DSR2-H349A-R	AGCACGAACAACAGTACCATTAACTTCAAA
DSR2-Y504A/K505A-F	GTCATGCTGCACCGTTTACCGATGAATTTTTAGC
DSR2-Y504A/K505A-R	TAAACGGTGCAGCATGACGACCAAAGGTTAACAATCC
DSR2-N548A-F	GAGTGATGCTCAATTTCTTTATGATGATACCGTAAAATT
DSR2-N548A-R	GAAATTGAGCATCACTCAGAAATTCCAGAATTTTGT
DSR2-Y574A/F576A/G577A-F	AGTGCTAGCGCCGCTATGTCATCTGATATTGTTGTACTGCTGC
DSR2-Y574A/F576A/G577A-R	CATAGCGGCGCTAGCACTGCCTTCACTCATTTCAGAACG
DSR2-H606A-F	CTGTGAGCTTTGCTGAATTTCATCAGTATATTCGTAATAGCATG
DSR2-H606A-R	TTCAGCAAAGCTCACAGACCACAGACAATTT
DSR2-N702A/G703A/M704A-F	GCTGCAGCAAACGTGGTCTTTTACACCCAGTTT
DSR2-N702A/G703A/M704A-R	ACCACGTTTGCTGCAGCCGCGCTAAACTGTTTGGTAATCT
DSR2-N961A-F	GTCATGGCTGAAAGCTTATAATGATAAACTGCTGGGTAAAATTG
DSR2-N961A-R	AAGCTTTCAGCCATGACGGAATAAATT

Table S1. Cont.

Primer name	Sequence
DSR2-N133A-F	GTTATCACCACTGCTTATGATAATTT
DSR2-N133A-R	TCAATTAAATTATCATAAGCAGTGGT
DSR2-H171A-F	TCTGCTTAAAGTTGCTGGCGATTTTC
DSR2-H171A-R	AATCGCCAGCAACTTTAAGCAGATAA
**TTP mutagenesis**	
TTP-S33A/I36A/R42A/I45A-F	CGAAGAAAAACTGGCTGGTGGTGCTGGCAATAAACCGCTGTATATTCTGAAAAGTGA
TTP-S33A/I36A/R42A/I45A-R	CAGCCAGTTTTTCTTCGCTAGCTGCCTGGGCAAAGCTGGCGGTCTGTGCT
TTP-K57A-F	CTGAAAAGTGAAGCAGAAATTAATCTGACCGTGAAAAATGC
TTP-K57A-R	TCTGCTTCACTTTTCAGAATATACAGCGGTT
TTPm2-F	GATATTGCTGCTGCGGCTGCGAATGTTAGCCCGAGCGGTG
TTPm2-R	AGCCGCAGCAGCAATATCGGCATACACTTCTTCGGTATCCGGATT
TTPm3-F	GATGAAGCGGCGGCGGCGATTGCAGCATCGCG
TTPm3-R	CGCGATGCTGCAATCGCCGCCGCCGCTTCATC
TTPm4-F	CACAGCTGCAGCAGCGAGCGCTGATCTGGGCGGCACCACG
TTPm4-R	TCGCTGCTGCAGCTGTGGCAGCCTCTACGGGGGCAGCCGT

Table S2. Statistics for cryo-EM data collection, refinement, and validation.

	**apo DSR2**	**DSR2-DSAD1**	**DSR2-TTP**	**intact DSR2-DSAD1**	**apo DSR2-local1**	**apo DSR2-loacal2**	**apo DSR2-loacal3**	**DSR2-DSAD1-local1**	**DSR2-DSAD1-local2**	**DSR2-TTP-local1**	**DSR2-TTP-local2**	**DSR2-TTP-local3**	**DSR2-TTP-local4**
**Data collection and processing**													
Microscope	Titan Krios G4	Titan Krios G4	Titan Krios G4	Titan Krios G4	Titan Krios G4	Titan Krios G4	Titan Krios G4	Titan Krios G4	Titan Krios G4	Titan Krios G4	Titan Krios G4	Titan Krios G4	Titan Krios G4
Camera	K3	K3	K3	K3	K3	K3	K3	K3	K3	K3	K3	K3	K3
Voltage (kV)	300	300	300	300	300	300	300	300	300	300	300	300	300
Electron exposure dose ($e^-/Å^2$)	48	60	48	48	48	48	48	60	60	48	48	48	48
Defocus range (μm)	0.7-1.7	0.7-1.7	0.7-1.7	0.7-1.7	0.7-1.7	0.7-1.7	0.7-1.7	0.7-1.7	0.7-1.7	0.7-1.7	0.7-1.7	0.7-1.7	0.7-1.7
Pixel size (Å)	0.65	0.65	0.65	0.65	0.65	0.65	0.65	0.65	0.65	0.65	0.65	0.65	0.65
Micrographs (total)	8,002	4,661	8,172	4,661	8,002	8,002	8,002	4,661	4,661	8,172	8,172	8,172	8,172
Micrographs (used)	5,643	3,680	10,825	3,680	5,643	5,643	5,643	3,680	3,680	10,825	10,825	10,825	10,825

Table S2. Cont.

	apo DSR2	DSR2-DSAD1	DSR2-TTP	intact DSR2-DSAD1	apo DSR2-local1	apo DSR2-loacal2	apo DSR2-loacal3	DSR2-DSAD1-local1	DSR2-DSAD1-local2	DSR2-TTP-local1	DSR2-TTP-local2	DSR2-TTP-local3	DSR2-TTP-local4
Final particle images (nos.)	141,731	269,385	102,635	42,050	141,731	141,731	141,731	269,385	269,385	102,635	102,635	102,635	102,635
Symmetry imposed	C1	C1	C1	C1	C1	C1	C1	C1	C1	C1	C1	C1	C1
Map resolution (Å)	2.92	2.98	3.16	6.99	3.25	2.92	2.81	2.93	2.96	3.10	2.91	2.98	3.21
FSC threshold	0.143	0.143	0.143	0.143	0.143	0.143	0.143	0.143	0.143	0.143	0.143	0.143	0.143
Map sharpening B factor ($Å^2$)	-71.4	-88.1	-76.0	-236.7	-87.3	-65.5	-63.7	-77.2	-90.3	-75.1	-73.4	-69.4	-73.2

Table S2. Cont.

	apo DSR2	DSR2-DSAD1	DSR2-TTP	intact DSR2-DSAD1	apo DSR2-local1	apo DSR2-loacal2	apo DSR2-loacal3	DSR2-DSAD1-local1	DSR2-DSAD1-local2	DSR2-TTP-local1	DSR2-TTP-local2	DSR2-TTP-local3	DSR2-TTP-local4
**Validation**													
MolProbity score	1.44	1.62	1.71	/	/	/	/	/	/	/	/	/	/
Poor rotamers (%)	0.46	0.49	0.87	/	/	/	/	/	/	/	/	/	/
Clashscore	3.94	4.41	5.48	/	/	/	/	/	/	/	/	/	/
RMS (bonds)	0.0067	0.0089	0.0122	/	/	/	/	/	/	/	/	/	/
RMS (angles)	1.15	1.24	1.29	/	/	/	/	/	/	/	/	/	/
Ramachandran plot													
Favored (%)	96.15	94.11	93.79	/	/	/	/	/	/	/	/	/	/
Allowed (%)	3.73	5.89	6.17	/	/	/	/	/	/	/	/	/	/
Disallowed (%)	0.12	0.00	0.19	/	/	/	/	/	/	/	/	/	/
